# Investigation on the Screening and Taste Mechanisms of Umami Peptides in Natural and Hydrolyzed Jinhua Ham

**DOI:** 10.3390/foods15112019

**Published:** 2026-06-04

**Authors:** Jing Cao, Yongxin Li, Hongang Tang, Huanhuan Li, Teng Xu, Ke Zhao, Jie Chen

**Affiliations:** 1 School of Food Science and Biotechnology, Zhejiang Gongshang University, Hangzhou 310018, China; 13732572087@163.com; 2 Zhejiang Key Laboratory of Intelligent Food Logistic and Processing, Institute of Food Science, Zhejiang Academy of Agricultural Sciences, Hangzhou 310021, China; 18247047851@163.com (Y.L.); zaastang@163.com (H.T.); huanhuanlee325@126.com (H.L.); 13031129533@163.com (T.X.)

**Keywords:** Jinhua ham, umami peptides, molecular docking, sensory evaluation, T1R1/T1R3

## Abstract

This study aimed to identify novel umami peptides from both natural and enzymatically hydrolyzed peptides from Jinhua ham, and to characterize their properties. Following LC–MS identification, known flavor sequences were annotated using a curated flavor-peptide database containing 2617 known flavor peptides, and potential novel umami peptides were screened through an integrated approach combining multiple machine learning models and LibDock molecular docking. From over 2000 identified peptides, this integrated virtual screening pipeline precisely yielded 10 candidate umami peptides, which were confirmed to possess distinct umami characteristics by sensory evaluation and electronic tongue analysis. These peptides had umami intensities ranging from 2.87 ± 1.38 to 3.90 ± 1.56 and significantly lower taste thresholds (0.1109–0.2634 mg/mL) than that of monosodium glutamate (MSG, 0.3 mg/mL). Subsequent CDOCKER analysis demonstrated significantly stronger binding affinity and stability of these peptides to the T1R1/T1R3 receptor compared with MSG, with -CDOCKER ENERGY values of 78.45–94.95 and -CDOCKER INTERACTION ENERGY values of 56.67–86.71, both significantly higher than those of MSG (22.27 and 46.58, respectively). Hydrogen bonds were identified as the dominant binding force, accounting for over 66% of total interactions, and HIS145 and ARG247 were confirmed as the key residues maintaining the stability of the peptide-receptor complex. Overall, these findings provide mechanistic insight into the umami properties of Jinhua ham peptides and support their potential application in food processing.

## 1. Introduction

Umami, one of the five basic tastes along with sour, sweet, bitter, and salty, has long been appreciated for its distinctive savory sensation and is considered essential for human nutrition [1]. The concept of umami was first introduced in 1908 by Kikunae Ikeda, who identified monosodium glutamate (MSG) as the principal compound responsible for the savory flavor of kelp broth. Umami peptides are a group of low-molecular-weight peptides (approximately 150–3000 Da) that impart umami taste characteristics. Owing to their natural origin, safety, nutritional value, and diverse bioactivities, they have emerged as an important focus in food science research [2]. In recent years, extensive studies have investigated umami characteristics of plant-derived protein hydrolysates, focusing on umami-active components, formation mechanisms and process optimization for natural flavor enhancers. Mu et al. [3] showed fermented soybean meal hydrolysates exhibit strong umami and umami-enhancing properties, driven by free glutamic acid/aspartic acid and small peptides (ME, EL, EA, EV). Peptides < 1000 Da dominate umami intensity, while 1000–3000 Da peptides mediate enhancement effects, with Aspergillus oryzae-derived aminopeptidase and glutaminase as key enzymes. Zhang et al. [4] identified six novel peanut umami peptides, including the first 14-residue umami-kokumi peptide with low thresholds (0.39–1.11 mM) and significant MSG synergism. Beyond their taste-modulating properties, such as enhancing umami perception or suppressing bitterness, umami peptides also exhibit various physiological functions. For instance, Hao et al. [5] reported that certain umami peptides possessed angiotensin-converting enzyme (ACE) inhibitory activity, while Zhang et al. [6] demonstrated that the peptide GPAGPAGPR showed dipeptidyl peptidase-IV (DPP-IV) inhibitory activity both before and after simulated gastrointestinal digestion in vitro.

Jinhua ham is one of China’s “Three Famous Hams” and is famous worldwide for its “Four Perfections”—color, aroma, taste, and form. During natural fermentation and maturation, endogenous proteases—including endopeptidases such as cathepsins B, D, H, and L, as well as calcium-activated proteases and exopeptidases like dipeptidases and aminopeptidases—continuously hydrolyze muscle proteins, generating abundant low-molecular-weight peptide fragments [7]. These naturally derived peptides play a pivotal role in shaping the characteristic flavor profile of Jinhua ham. Among them, certain short peptides exhibit potential umami activity and are considered major contributors to its distinctive taste [8]. In addition to naturally fermented peptides, enzymatic hydrolysis serves as an effective approach for producing umami peptides, which could yield structurally diverse peptides with rich flavor potential from protein hydrolysates [9].

To date, research on Jinhua ham peptides has mainly focused on their antioxidant activities, while other studies have investigated antibacterial [10], enhance saltiness [11], and anti-inflammatory [12] effects. However, research specifically targeting umami peptides from Jinhua ham remains very limited. Notably, Dang et al. [8] reported the identification of an umami peptide, Cys-Cys-Asn-Lys-Ser-Val, from the water-soluble extract of Jinhua ham. At the same time, no studies have systematically explored novel umami peptides from the perspectives of both naturally occurring peptides and enzymatically generated peptides during fermentation. Therefore, a comprehensive investigation of Jinhua ham umami peptides and their underlying mechanisms is of significant industrial and scientific importance.

Conventional approaches for umami peptide screening generally involve multiple steps, including proteolytic digestion, separation and purification, and mass spectrometric identification [13]. These procedures are often labor-intensive and time-consuming, resulting in low screening efficiency. Consequently, computer-aided techniques such as machine learning and molecular docking have attracted increasing attention for their ability to accelerate the discovery and identification of umami peptides [14].

Molecular docking is a computational technique used to simulate the binding interactions between peptide fragments and target proteins [15]. By assessing geometric complementarity and interaction energies, it predicts the stability of peptide–protein complexes and facilitates the screening of potential candidate molecules [16]. Among the identified umami receptors, T1R1/T1R3 are recognized as the primary umami receptors in humans. Their three-dimensional structures are commonly obtained through homology modeling, after which potential umami peptides are docked to evaluate binding affinity and stability, thereby elucidating the molecular mechanisms underlying peptide–receptor interactions [17]. In recent years, an increasing number of studies have utilized molecular docking to elucidate the umami mechanisms of protein hydrolysates. For example, previous studies have applied this technology to identify key umami peptides from various food matrices, revealing how specific amino acid residues (such as glutamic acid, aspartic acid, and hydrophobic residues) strongly interact with the active pockets of the T1R1/T1R3 receptor via hydrogen bonds and hydrophobic interactions [18,19]. Concurrently, advanced machine learning models such as Umami-MRNN, Umami-YYDS, and TPDM extract informative features from high-quality peptide datasets to construct predictive frameworks, enabling rapid and accurate evaluation of umami potential during high-throughput screening. These computational approaches substantially shorten the experimental validation cycle, thereby improving the efficiency of candidate umami peptide screening and optimization. Ultimately, the predicted umami peptides are subjected to sensory evaluation and electronic tongue analysis to verify their umami activity.

Therefore, to further elucidate umami peptides in Jinhua ham and its potential flavor components, this study combined protease hydrolysis with naturally fermented peptides to obtain a broader range of candidate umami peptides. The novelty of the present study lies in the first systematic exploration of umami peptides from both naturally occurring and enzymatically generated perspectives during the fermentation of Jinhua ham. By establishing a closed-loop research framework that integrates data analysis, machine learning-based prediction, molecular docking screening, and sensory as well as docking validation, this work systematically and efficiently identified both natural and enzyme-derived umami peptides from Jinhua ham. The proposed approach provides new theoretical insights and technical support for advancing umami peptide research in traditionally fermented meat products.

## 2. Materials and Methods

### 2.1. Reagents and Materials

One-year-aged Jinhua ham, produced from Du Changbai three-way crossbred pigs, was obtained from Jinhua Jinnian Ham Co., Ltd. (Jinhua, China). Trypsin powder was purchased from Nanning Pangbo Biological Engineering Co., Ltd. (250,000 U/g, Nanning, China). Chromatography-grade solvents were used for chromatographic analyses, and all other chemicals were of analytical grade. Sucrose, monosodium glutamate, edible salt, and food-grade citric acid were purchased from local supermarkets. Quinine was supplied by Xi’an Feida Biotechnology Co., Ltd. (Xi’an, China). The target peptide was synthesized by Nanjing Peptide Research Biotechnology Co., Ltd. (Nanjing, China) with a purity > 95%.

### 2.2. Sample Preparation

Natural peptides (Nat group) were extracted to represent endogenous peptides naturally released during ham ripening, following the method described by Escudero et al. [20] with minor modifications. Briefly, after removing fat and connective tissue, 25 g of minced ham was mixed with 100 mL of 0.02 M phosphate-buffered saline (PBS, pH 7.0). The mixture was homogenized using a high-speed tissue homogenizer at 12,000 rpm for 10 min until a homogeneous viscous slurry was obtained. The homogenate was incubated at 4 °C for 2 h and subsequently centrifuged at 12,000× *g* for 20 min. The resulting supernatant was collected, mixed with three volumes of 70% (*v*/*v*) ethanol, and kept at 4 °C for 8 h, followed by another round of centrifugation for 20 min. Finally, crude peptide fractions were obtained via rotary evaporation and lyophilization, and stored at −20 °C for further analysis.

Enzymatic peptides (Enz group) were prepared to expand the identification of potential umami peptide sequences beyond those naturally generated in ham. Based on our laboratory-optimized protocol for maximizing peptide yield and structural diversity, trypsin was selected as the optimal hydrolytic enzyme from several candidates [21]. Briefly, 100 g of lean Jinhua ham meat was blended with 200 g of water and homogenized at 12,000 rpm for 10 min using a tissue homogenizer to form a uniform viscous slurry. The homogenized lean ham sample was dialyzed against distilled water (1:100, *v*/*v*) to remove salts prior to enzymatic hydrolysis. Hydrolysis was carried out with trypsin at an enzyme-to-substrate ratio of 1600 U/g under conditions of pH 8.0 and 37 °C for 5 h. The enzymatic reaction was terminated by boiling for 10 min, and the supernatant was collected for subsequent identification of novel umami peptides.

### 2.3. LC-MS Identification and Trapping

For LC-MS analysis, mobile phase A was 0.1% formic acid in water, and mobile phase B was 0.1% formic acid in 84% aqueous acetonitrile. The liquid chromatography column (0.15 mm × 150 mm, RP-C18, Column Technology Inc., Fremont, CA, USA) was equilibrated with 95% mobile phase A. Samples were loaded via an autosampler onto Zorbax 300SB-C18 peptide traps (Agilent Technologies, Wilmington, DE, USA) and subsequently separated on the LC column. The gradient program was as follows: 0–50 min, mobile phase B increased linearly from 4% to 50%; 50–54 min, B increased to 100%; 54–60 min, B held at 100%. After mass spectrometry separation, QExactive mass spectrometry (Thermo Fisher, Waltham, MA, USA) was used for mass spectrometry analysis. Analysis duration: 60 min. Detection mode: positive ions.

Raw mass spectrometry data were analyzed using MaxQuant 1.5.5.1 against relevant databases with the following parameters: Enzyme: None; Max Missed Cleavages: 2; Primary ion mass tolerance: 20 ppm; MS/MS tolerance: 0.1 Da; FDR threshold: ≤0.01. Following Zhang et al. [22], only peptides with molecular weights < 1500 Da were selected for subsequent analysis due to their stronger umami characteristics.

### 2.4. Database Construction

To investigate the presence of unknown umami peptides and the distribution of known flavor peptides in Jinhua ham, a comprehensive flavor peptide database was constructed by integrating datasets from four predictive models and four existing peptide databases. The predictive models included UmamiBERT (https://github.com/katouMegumiH/Umami.github.io, accessed on 11 January 2025), UmamiMRNN (https://umami-mrnn.herokuapp.com, accessed on 11 January 2025), iUmami SCM (http://camt.pythonanywhere.com/iUmami-SCM, accessed on 11 January 2025), and UmamiGCForest (http://www.syaufoodumami.online, accessed on 11 January 2025). The peptide databases comprised TastePeptidesDB (http://tastepeptides-meta.com/TastePeptidesDB, accessed on 20 January 2025), BIOPEP (https://biochemia.uwm.edu.pl/biopep/start_biopep.php, accessed on 20 January 2025), the Shanghai Jiao Tong University Flavor Database (SJDDB, https://mffi.sjtu.edu.cn/database, accessed on 20 January 2025), and BTD640 (https://github.com/Shoombuatong/Dataset-Code/tree/master, accessed on 20 January 2025).

Following the method of Cui et al. [23], collected peptides were filtered as follows: (1) only sequences containing 2–25 amino acids were retained; (2) sequences containing non-standard amino acid codes (e.g., B, U, X, Z) were removed; (3) redundant sequences within the same flavor category were eliminated; and (4) the remaining peptides were annotated with seven flavor attributes: sour, sweet, bitter, salty, umami, kokumi, and astringent. The curated flavor peptide database was then compared with the ham peptides identified in this study to determine the distribution of known flavor peptides and to identify potential novel umami peptides.

### 2.5. Toxicity and Umami Peptides Prediction

Following Zhu et al. [24] with slight modifications, unknown flavor peptides identified through database comparison were first screened for potential toxicity using ToxinPred 3.0 (https://webs.iiitd.edu.in/raghava/toxinpred3/prediction.php, accessed on 6 June 2025). The intersection of three independent umami prediction models was then used to define the target peptide set: Umami-YYDS (http://tastepeptides-meta.com/Umami_YYDS, accessed on 6 June 2025), trained on 84 umami and 129 non-umami peptides using eight selected features and a Gradient Boosting classifier; Umami-MRNN (https://umami-mrnn.herokuapp.com), trained on 212 umami and 287 non-umami peptides with six sequence features and a hybrid MLP-RNN architecture; and TPDM (http://tastepeptides-meta.com/TPDM, accessed on 6 June 2025), trained on 244 umami and 257 non-umami peptides using five machine learning algorithms combined with four molecular representations.

### 2.6. Homology Modeling of the Umami Receptor T1R1/T1R3 Protein Dimer

For ligands, three-dimensional structures of detected ham peptides were generated using RDKit following Yu et al. [25]. As no crystal structure of the human umami receptor T1R1/T1R3 is available, homology modeling was performed based on Song et al. [26]. Amino acid sequences of T1R1 (UniProt ID: Q7RTX1) and T1R3 (UniProt ID: Q7RTX0) were retrieved from UniProtKB (https://www.uniprot.org/). The heterodimer model was constructed using SWISS-MODEL (https://swissmodel.expasy.org) with the template showing the highest sequence identity (PDB ID: 5X2Q, 37.6%). The resulting structure was saved in PDB format and evaluated using the Profiles-3D module in Discovery Studio and Ramachandran analysis with PROCHECK on the SAVES v6.1 platform (https://saves.mbi.ucla.edu/).

### 2.7. LibDock Molecular Docking Screening

Prior to docking, multiple conformations of ham peptide ligands were generated using the “Prepare Ligands” module in Discovery Studio “Small Molecules.” The T1R1/T1R3 receptor heterodimer was preprocessed by removing water molecules and pre-bound ligands, adding hydrogens, and defining the active site. LibDock docking was performed with “Docking Preferences” set to “User Specified” and “Max Hits to Save” set to 5, while all other parameters were kept at default.

### 2.8. Sensory Evaluation

The sensory assessment was approved by the Ethics Committee of Zhejiang Gongshang University and conducted at 24 ± 2.5 °C in the university’s Sensory Laboratory. The panel consisted of 10 trained subjects (5 males, 5 females, 22–30 years old) with no history of smoking, excessive alcohol consumption, or taste disorders, who provided written informed consent. Panelists were trained to recognize the five basic tastes (sour, sweet, bitter, salty, and umami). To prevent bias, all synthetic peptide samples were labeled with randomly generated three-digit codes.

Sensory intensity was evaluated following Pan et al. [2]. Five reference solutions, 0.35% MSG (umami), 0.08% citric acid (sour), 0.08% quinine (bitter), 0.35% sodium chloride (salty), and 1% sucrose (sweet), were assigned an intensity of 5. Synthetic peptides were dissolved in ultrapure water (1 mg/mL) and adjusted to pH 6.5. Participants avoided eating, smoking, or exposure to strong odors for at least 2 h prior to testing. Samples were presented sequentially, held in the mouth for ~10 s, and rated on a 0–10 scale (0 = undetectable, 10 = extremely strong). To minimize carryover effects, panelists rinsed with ultrapure water three times and waited 1 min between samples. The taste threshold of synthetic peptides was determined using the three-point forced-choice (3-AFC) method. Peptide solutions were prepared in a twofold dilution series (1, 0.5, 0.25, 0.125, and 0.0625 mg/mL) using deionized water.

### 2.9. Electronic Tongue

The flavor profiles of synthetic peptides were evaluated using an SA402B electronic tongue (Insent Corporation, Atsugi, Japan) following Tang et al. [27]. Peptides were tested at 0.2 mg/mL, with MSG at the same concentration as a control. The electronic tongue was equipped with five sensors (C00, AE1, CA0, CT0, AAE) and two standard electrodes, and 30 mM KCl and 0.3 mM tartaric acid was used as reference solution. Electrodes were cleaned using negative (100 mM HCl, 30% ethanol) and positive (10 mM KOH, 100 mM KCl, 30% ethanol) solutions. Each measurement consisted of a balancing phase (rinsing and reference calibration) followed by a 30 s detection and 30 s aftertaste test. Samples were measured in four replicates, discarding the first, and the mean of the remaining three was used for analysis.

### 2.10. CDOCKER Molecular Docking

CDOCKER handles ligands and receptors in a manner similar to LibDock, with the “Pose Cluster Radius” set to 0.5 and all other parameters kept at default. Binding affinities and receptor-interacting residues were analyzed using the “Analyze Ligand Poses” module in Discovery Studio. In the docking results, The -CDOCKER ENERGY (-CE) score reflects overall binding stability, including both the ligand’s internal conformation energy and ligand–receptor interaction energy, with higher values indicating greater stability.

### 2.11. Statistics and Analysis

All experiments were performed in triplicate, and results are expressed as mean ± standard deviation (SD). Statistical significance was evaluated by one-way ANOVA followed by Tukey’s post-hoc test using Prism 10.1.2 (GraphPad Software, Santiago, CA, USA), with *p* < 0.05 considered significant. Electronic tongue data were autoscaled and subjected to principal component analysis (PCA) using Origin 2021 (OriginLab, Northamption, NC, USA). Data visualization (including the PCA score plot) was performed using Origin 2021 and Matplotlib 3.9.2. Molecular docking figures were generated with Discovery Studio v19.1.0 and PyMOL 3.0.

## 3. Results and Discussion

### 3.1. Identification and Analysis of Ham Peptides

**Figure 1:**
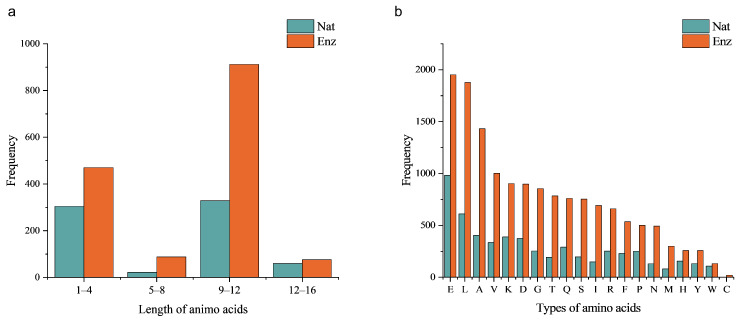
LC-MS identification analysis. (**a**) Peptide length distribution in the Nat and Enz groups. (**b**) Amino acid composition distribution in the Nat and Enz groups.

A total of 716 and 1698 peptide fragments were identified in the Nat group and Enz group, respectively, indicating that enzymatic hydrolysis markedly enhanced protein degradation and peptide release. The peptide length distribution (Figure 1a) exhibited a bimodal pattern, primarily within 3–5 and 10–12 amino acid residues. This pattern likely reflects both enzymatic cleavage preferences and protein structural constraints, suggesting that umami peptides in Jinhua ham may potentially enrich within these two length ranges.

Given the strong link between amino acid composition and taste, further analysis regarding the amino acids constituents of the detected peptides was performed (Figure 1b). The predominant residues in both groups were glutamic acid (E), leucine (L), alanine (A), valine (V), lysine (K), and aspartic acid (D), accounting for 56.1% and 53.5% of total amino acids in the Nat and Enz groups, respectively. Among these, E and D are recognized as umami contributors, whereas L, V, and K are generally associated with bitterness [28]. After enzymatic hydrolysis, the absolute content of umami amino acids increased from 1356 to 2848 (a 2.10-fold increase), but it remained lower than that of bitter amino acids, which increased from 1333 to 3781 (a 2.83-fold increase). Consequently, the proportion of umami amino acids in the total amino acid content decreased from 24.6% to 18.9%. These results are consistent with previous studies indicating that proteolysis tends to enhance bitterness [29]. Overall, the combination of short peptide enrichment and high levels of umami-associated residues highlights taste potential, providing a solid basis for targeted screening of umami-active peptides from Jinhua ham.

### 3.2. Analysis of Flavor Peptides in Jinhua Ham

**Figure 2:**
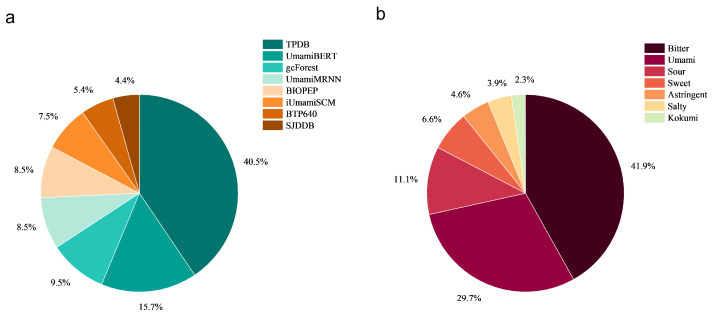
Source and distribution of flavor peptides. (**a**) Source distribution of known flavor peptides in the peptide library. (**b**) Flavor distribution of known peptides after redundancy removal under a single flavor classification.

To elucidate the composition of flavor peptides in Jinhua ham and identify potential novel umami peptides, a comprehensive database comprising 5861 flavor peptides was established. The peptides in the database ranged from 2 to 25 amino acid residues in length. Specifically, the entries were derived from TPDB (2376), UmamiBERT (921), gcForest (558), UmamiMRNN (498), BIOPEP (496), iUmamiSCM (440), BTP640 (317), and SJDDB (255) (Figure 2a). Due to the overlap of peptide entries across different source databases, redundant sequences were removed to ensure data integrity. Each unique sequence was then systematically categorized, yielding 2617 non-redundant entries. The flavor distribution of these peptides was as follows: bitter (1096), umami (776), sour (291), sweet (172), astringent (120), salty (102), and kokumi (60) (Figure 2b). Bitter and umami peptides notably dominated the dataset.

**Figure 3:**
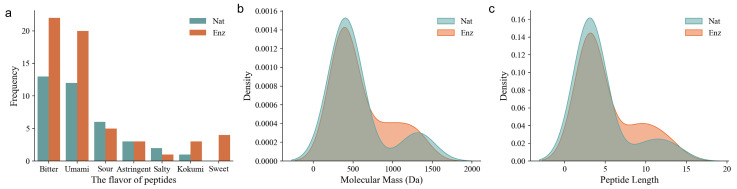
The composition of flavor peptides in Jinhua ham and the molecular weight distribution and tryptophan length distribution of its umami peptides are known. (**a**) Flavor distribution of known peptides in Nat and Enz groups. (**b**) Molecular weight distributions of known umami peptides in the Nat and Enz groups. (**c**) Peptide length distributions of known umami peptides in the Nat and Enz groups.

Alignment of peptide sequences from the Nat and Enz groups against the flavor peptide library identified 26 and 40 known peptides (Figure 3a, Table S1), respectively. Bitter and umami peptides were the predominant flavor types in both groups, collectively accounting for more than 65%, suggesting that these two categories largely define the flavor profile of Jinhua ham. Among them, 12 and 20 peptides were classified as umami peptides, including six shared sequences (DEL, EEL, LDF, LEF, TEF, and EQYEEEQEAK). Further analysis revealed that umami peptides from both groups exhibited similar molecular weight distributions and sequence lengths, with most majority of them owing 300 to 500 Da molecular weights and 3–5 amino acids residual (Figure 3b,c). These observations indicate that short-chain peptides (300–500 Da, 3–5 residues) constitute the predominant structural form of umami peptides, consistently observed across both Nat and Enz.

### 3.3. Toxicity and Umami Prediction for Unknown Peptides

**Figure 4:**
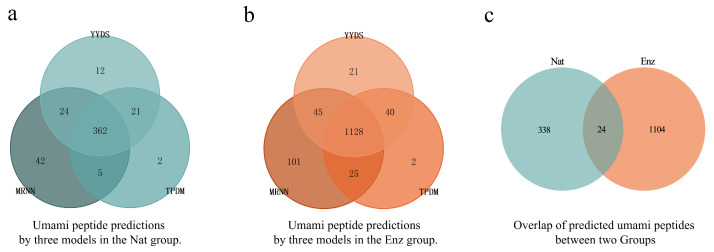
Venn diagrams of predicted umami peptides in the Nat and Enz groups based on three models. (**a**) Venn diagram of three umami peptide prediction models in the Nat group; (**b**) Venn diagram of three umami peptide prediction models in the Enz group; (**c**) Overlap of predicted umami peptides shared between the Nat and Enz groups.

After excluding those known flavor peptides mentioned above, 690 and 1658 unknown flavor peptides were retained in the Nat and Enz groups, respectively. Toxicity prediction yielded 528 and 1471 non-toxic candidates in the Nat and Enz groups, respectively. These peptides were subsequently evaluated using three independent predictive models of umami peptides (Umami-YYDS, Umami-MRNN, TPDM), and 362 and 1128 peptides were consistently predicted as umami peptides in the Nat and Enz groups (Figure 4a,b), respectively. Notably, the number of predicted umami peptides in the Enz group was substantially larger than that in the Nat group, suggesting that enzymatic hydrolysis potentially generates a richer repertoire of umami peptides. Further comparison between the two groups (Figure 4c) revealed only 24 overlapping umami peptides (Table S2), indicating marked compositional differences between the two sources. This finding suggests that the Nat and Enz groups possess distinct umami profiles that may contribute complementary flavor characteristics to Jinhua ham. Overall, the integrated strategy combining toxicity screening with multi-model intersection effectively identified 362 non-toxic umami peptide candidates from the Nat group and 1128 from the Enz group, while the umami potential of those peptides require further validation.

### 3.4. Homology Modeling

**Figure 5:**
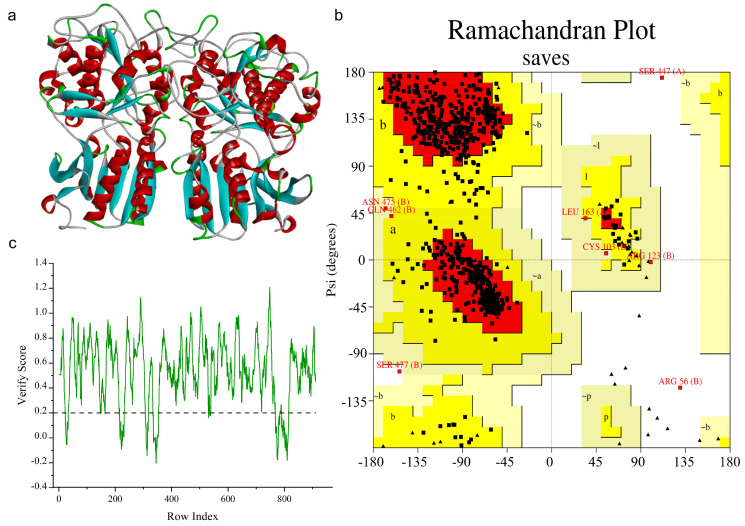
Modeling and validation of the umami receptors T1R1/T1R3. (**a**) Three-dimensional structural model of the T1R1/T1R3 receptors; (**b**) Ramachandran plot of the T1R1/T1R3 receptors; (**c**) Amino acid Verify Score plot of the T1R1/T1R3 receptors.

To further evaluate those umami peptide candidates predicted above, a three-dimensional structural model of T1R1/T1R3 receptor dimer was generated using SWISS-MODEL (Figure 5a). DS Profiles-3D analysis yielded a Verify Score of 449.19 (Figure S1), which was substantially higher than both the Verify Expected Low Score (190.916) and the Verify Expected High Score (424.258), confirming the structural validity and reliability of the model [30]. Further evaluation with the PROCHECK module of the SAVES platform showed that 88.5% of residues in the Ramachandran plot (Figure 5b) were located in the core preferred region, 10.5% in the allowed region, and only 0.4% in the disallowed region. The combined proportion of residues in the core and allowed regions reached 99% (>90%), indicating good geometric rationality and conformational stability of the model in terms of dihedral angle distribution [31]. In addition, a scatter plot of the Amino Acid Verify Score from Profiles-3D (Figure 5c) demonstrated that 88% (>80%) of amino acids achieved a 3D/1D score ≥ 0.2, further validating the accuracy of the umami receptor model. As demonstrated above, this model is suitable for subsequent molecular docking studies.

### 3.5. Libdock Screening

After the initial screening based on database comparison, toxicity evaluation, and umami prediction, potential umami peptides were further identified through molecular docking using LibDock. Using successful docking results and the LibdockScore of glutamic acid (71.55) as screening criteria, 12 out of 363 potential umami peptides in the Nat group were able to bind to the active sites of the T1R1/T1R3 receptor. Similarly, 23 of the 1128 potential umami peptides in the ENZ group (Table 1). Docking analysis revealed that the lengths of the 35 peptides were mainly concentrated in the range of 3 to 4 amino acid residuals, consistent with previous findings [29] and the findings in Section 3.2 that most known umami peptides in Jinhua Ham comprise 3 to 5 amino acids, with molecular weights between 300 and 500 Da. The type and composition of amino acids within peptides are generally closely associated with their sensory properties. Among them, glutamic acid (E) and aspartic acid (D) residues typically contribute to umami perception. It was found that approximately 75% of the 35 umami peptides candidates contained more than 25% umami-related residues (E, D), suggesting that the majority of candidate may merit umami flavor.

The LibDockScores of the 35 umami candidate peptides ranged from 73.87 to 152.50, with EGY, LDAL, and EQY exhibiting the highest scores. Comparison of the Enz and Nat groups revealed that the Enz group peptides were mainly distributed within the range of 113.19–132.84, accounting for 47.8% of the total, with 13.1% falling in the high-score range (132.84–152.50). In contrast, the Nat group peptides were predominantly distributed within 93.53–113.19 (41.7%), and only 8.3% appeared in the high-score range. Overall, the Enz group displayed a higher score distribution, indicating stronger receptor affinity potential, while the Nat group tended to cluster within the mid-to-low score range, though individual peptides such as LDAL also exhibited notably high affinity.

Analysis of the 35 candidate peptides revealed concentrated distributions across the three predictive models (Figure S2), with mean/median values of 0.97/1.00 (YYDS), 0.95/0.98 (TPDM), and 17.22/14.38 (MRNN). Given that LibDockScores primarily reflect binding affinity rather than direct sensory umami intensity, we established stringent thresholds (TPDM > 0.97, YYDS > 0.99, and MRNN < 15) based on the methodology of Yu et al. [32] and the aforementioned statistical patterns to minimize false positives. This strategy prioritized candidates demonstrating consistently robust and superior performance across all algorithmic models, leading to the selection of 13 high-confidence umami peptides (marked with *) for subsequent experimental validation.

### 3.6. Sensory Testing and Electronic Tongue Results

**Figure 6:**
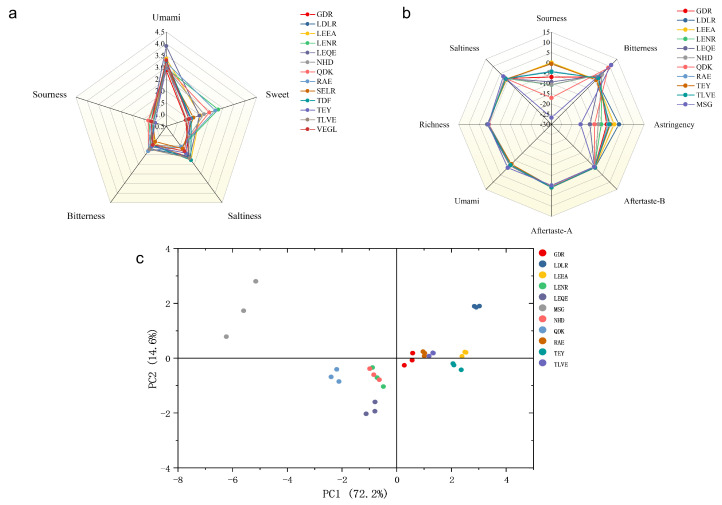
Sensory and electronic tongue analysis. (**a**) Sensory evaluation radar chart. (**b**) Electronic tongue radar chart. (**c**) PCA chart based on electronic tongue data.

The taste of the 13 umami peptide candidates were further evaluated through sensory testing and electronic tongue analysis. The results showed that the 13 peptides exhibited distinct umami characteristics with varying umami intensities (Figure 6a), and the taste scores ranging from 2.87 to 3.90. Among them, LEQE showed the strongest umami perception (3.90 ± 1.56), followed by LEEA (3.37 ± 1.79), whereas GDR presented the weakest (2.87 ± 1.38). The overall ranking of umami intensity was as follows: LEQE > LEEA > VEGL > TDF > RAE > LDLR > SELR > TLVE > QDK = TEY > LENR = NHD > GDR. The intense umami perception of LEQE and LEEA can be directly attributed to their specific amino acid compositions. Both peptides contain multiple glutamic acid (Glu, E) residues, which are universally recognized as the core driving force for umami taste due to the interaction of their side-chain carboxyl groups with umami receptors [33]. Conversely, peptides with relatively lower umami scores, such as GDR and NHD, contain fewer typical umami amino acids and are rich in basic (Arg, R) or amide-containing (Asn, N) residues, which may impart complex taste profiles that dilute pure umami perception [4]. In terms of flavor composition, all 13 peptides were predominantly characterized by umami as the dominant taste attribute, accompanied by secondary notes of saltiness and sweetness, while bitterness remained mild and sourness consistently the weakest among the five basic tastes.

To systematically evaluate the umami potential of 13 peptide segments, sensory threshold measurements were conducted. After screening, 3 peptides with abnormal threshold values were excluded, and the remaining 10 were identified as new umami peptides from Jinhua ham exhibiting distinct umami characteristics (Figure 6b). Ranked from lowest to highest, the thresholds were: NHD < LENR < QDK < LDLR < TEY < LEEA < TLVE < LEQE < GDR < RAE. The threshold range was 0.1109–0.2634 mg/mL, all lower than that of MSG (0.3 mg/mL) [34], indicating that the umami taste of these peptides is more readily perceived than that of MSG. To further interpret the significance of the identified peptides, their sensory characteristics were compared with those reported in previous studies on other food matrices. For instance, the umami peptide ALDELGT identified from soybean paste exhibited a threshold of 1.45 mg/mL [2], and umami peptides from fermented grain wine (Huangjiu) typically range from 0.13 to 0.87 mg/mL [35]. This underscores the exceptional flavor-enhancing potential of Jinhua ham peptides.

Principal component analysis (PCA) of the electronic tongue data revealed a clear separation between MSG and the 10 candidate peptides (Figure 6c), indicating distinct differences in umami perception. Notably, although QDK and LEQE displayed radar chart profiles similar to MSG, they did not cluster with MSG in PCA, suggesting that these peptides share localized taste similarities but differ substantially in overall taste characteristics. Other peptides exhibited varying degrees of aggregation: RAE, TLVE, and GDR clustered near the origin, while LENR and NHD, as well as TEY and LEEA, formed separate clusters.

**Figure 7:**
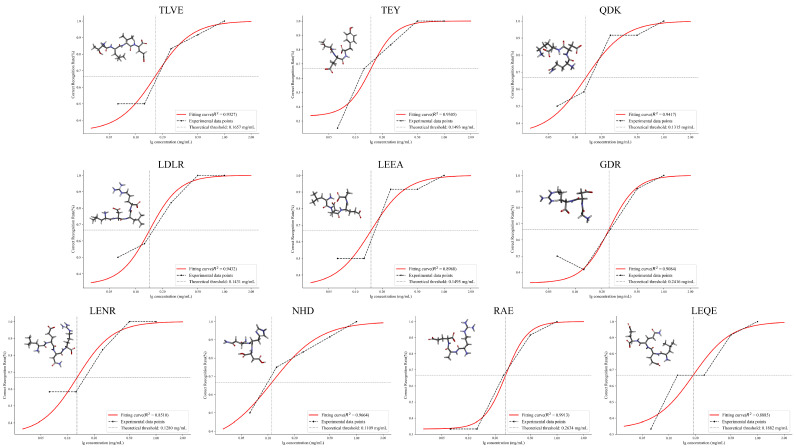
Threshold fitting curve of 10 umami peptides.

Subsequently, the taste characteristics of these ten umami peptides were further verified and compared using electronic tongue analysis (Figure 7); all candidate peptides exhibited performance comparable to MSG in umami, saltiness, richness, aftertaste-A, and aftertaste-B, while slightly exceeding MSG in sourness and astringency, which may be attributed to residual trifluoroacetic acid from peptide synthesis [36]. In contrast, all peptides showed significantly reduced bitterness compared with MSG, likely because the peptide chain structure partially masked internal hydrophobic amino acid residues, thereby lowering bitterness perception [37].

The results of the electronic tongue and sensory evaluations revealed both consistencies and discrepancies. Consistency was mainly reflected in the observation that acidity represented the weakest taste attribute across all ten candidate peptides in both methods, and that all peptides exhibited a certain degree of saltiness, confirming their characteristic profile of “low acidity with mild saltiness.” However, differences were evident in the perception of umami and bitterness. The umami intensity that was prominent in sensory evaluation did not correspond to strong responses in the electronic tongue analysis (Sensory umami: 2.87 to 3.90; E—tongue umami: −2.53 to −1.45). In contrast, bitterness-perceived as relatively mild in sensory testing produced stronger response signals in the electronic tongue measurements (Sensory bitterness: 1.37 to 1.80; E—tongue bitterness: 0.66 to 8.92). According to previous studies, this discrepancy does not indicate methodological bias but rather reflects inherent differences in perceptual mechanisms [38]. Specifically, the electronic tongue assesses umami solely by detecting electrical responses generated by taste-active molecules through specialized sensors, whereas sensory evaluation relies on a series of physiological processes [39].

Regarding the origin of bitterness, particularly in the enzymatically generated peptides (Enz group), the specific cleavage mechanism of the enzyme plays a crucial role. Trypsin specifically cleaves peptide bonds at the carboxyl side of basic amino acids, namely arginine (Arg, R) and lysine (Lys, K) [40], which explains why enzymatic peptides such as LDLR, QDK, GDR, and LENR all possess basic amino acids at their C-termini. While previous studies suggest that basic amino acids at the C-terminus can enhance umami perception through specific spatial conformations [41], they are also well-known contributors to bitterness. In our sensory evaluation, this intrinsic bitterness was likely masked by the strong umami signals from adjacent acidic amino acids (like Asp and Glu) via taste–taste interaction (umami masking bitterness) [42]. However, the electronic tongue, free from physiological masking effects, objectively detected the exposed hydrophobic and basic groups, resulting in the higher bitterness response signals observed.

### 3.7. CDOCK Molecular Docking Results

**Figure 8:**
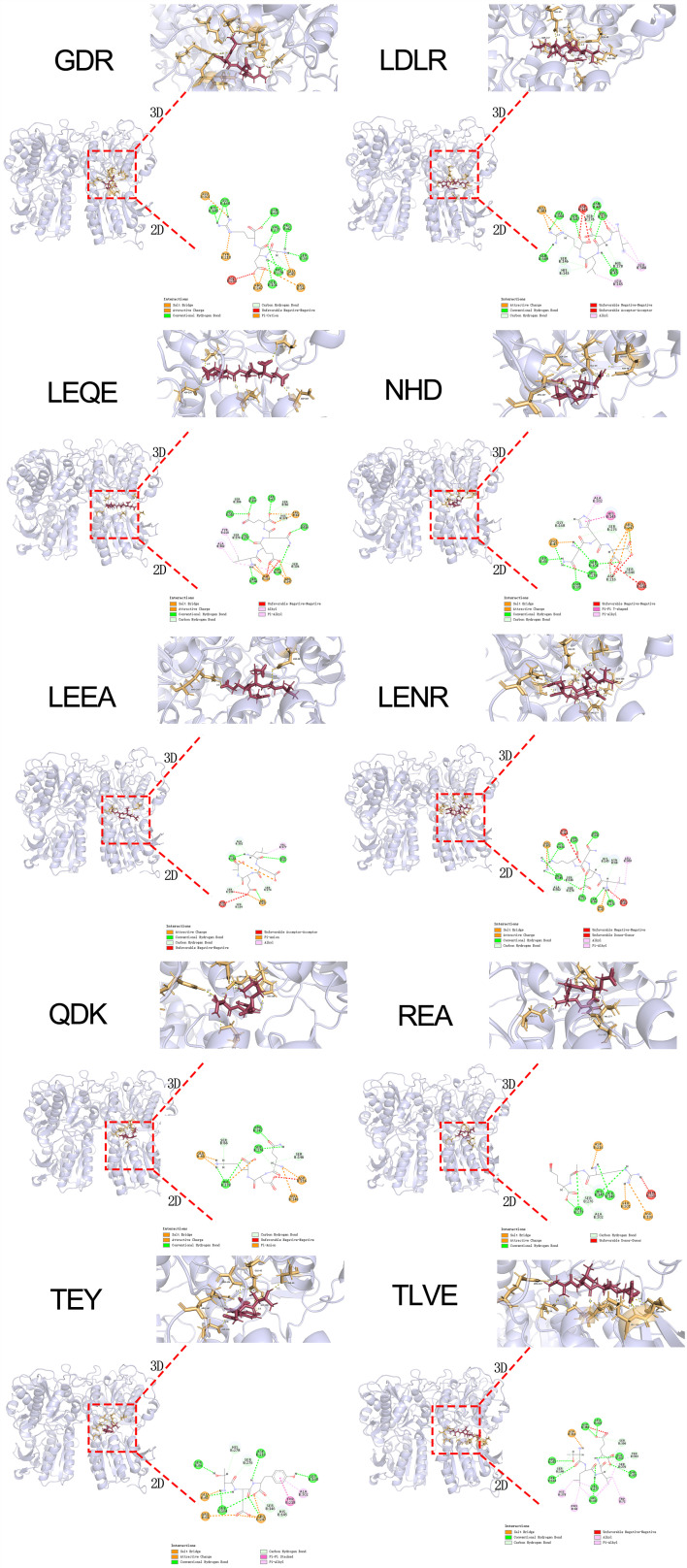
Three-dimensional and two-dimensional docking diagrams of umami peptides with umami receptors T1R1/T1R3.

The key step in umami perception is the binding of umami peptides to the T1R1/T1R3 receptor complex [43]. Molecular docking provides insights into ligand–receptor interaction patterns by exploring their optimal binding conformations. To investigate the binding modes of the selected umami peptides with T1R1/T1R3, docking analysis was performed using the CDOCKER module in Discovery Studio. The 2D and 3D schematic representations of the docking results for the 10 target umami peptides with T1R1/T1R3 are shown in (Figure 8).

The docking analysis revealed that the -CDOCKER ENERGY values of the ten umami peptides ranged from 75.41 to 96.35, while the -CDOCKER INTERACTION ENERGY values ranged from 56.67 to 86.72. Both parameters were markedly higher than those of the control compound MSG (22.27 and 46.58, respectively), suggesting that these peptides possess stronger receptor affinity, enhanced binding stability, and greater potential umami activity. Among them, QDK displayed the highest -CDOCKER ENERGY values 96.35.

A combined analysis of the molecular docking and sensory analyses revealed that umami peptides with higher receptor affinity (relative to MSG) generally exhibited lower perceptual thresholds. For instance, QDK, LEQE, and LENR exhibited relatively high -CDOCKER ENERGY values (>90) and low perceptual thresholds (<0.15 mg/mL), implying a correlation between umami perception capacity and binding affinity to T1R1/T1R3 receptors. However, this relationship was not reflected in umami intensity. For example, although QDK demonstrated higher affinity than LDLR, it produced lower umami scores in both sensory evaluation and electronic tongue assays. This observation is consistent with previous reports suggesting that receptor affinity alone is insufficient to fully determine the perceived intensity of umami [44].

**Figure 9:**
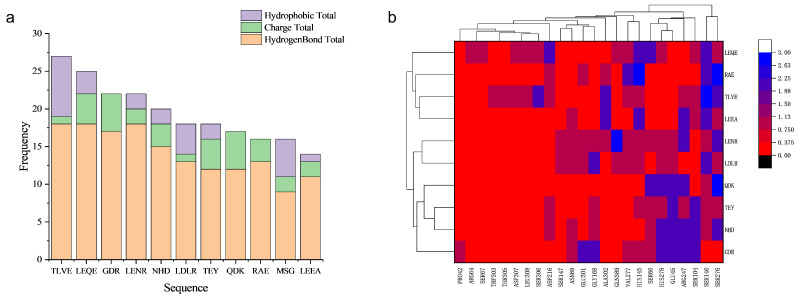
The CDOCKER docking forces between 10 umami peptides and umami receptors T1R1/T1R3. (**a**) Overlay of binding forces between ten peptides (MSG as positive control) and the T1R1/T1R3 receptor. (**b**) Heatmap of hydrogen bond interactions between 10 peptides and umami receptors T1R1/T1R3.

Previous studies have demonstrated that the stable binding of umami peptides to the T1R1/T1R3 receptor complex primarily depends on three major types of interactions: hydrogen bonding (including conventional and carbon–hydrogen bonds), electrostatic interactions (such as salt bridges and charge attraction), and hydrophobic interactions [45]. Analysis of the binding force composition between the ten umami peptides and T1R1/T1R3 (Figure 9a, Table S3) revealed that hydrogen bonding was the predominant interaction, consistent with earlier reports [44]. For all ten peptides, hydrogen bonds accounted for more than two-thirds of the total binding forces. Among them, LEQE, LENR, and TLVE exhibited the highest number of hydrogen bonds (18 each), whereas LEEA formed the fewest (11). Meanwhile, as shown in Figure 9b, the key receptor residues involved in hydrogen bond formation were SER276 (16), SER146 (15), HIS145 (12), and ARG247 (12).

Although electrostatic and hydrophobic interactions accounted for less than one-third of the total binding force, they remained essential for maintaining the stability of the peptide–receptor complex. As shown in Table 2, TLVE and LDLR exhibited pronounced hydrophobic interactions with receptor residues such as HIS145, VAL277, and ALA302. This may be attributed to the abundance of hydrophobic amino acids (e.g., Leu, Val) in these peptides, which allows them to embed into hydrophobic regions of the receptor (e.g., VAL277, ALA302, TYR218, TRP72), thereby forming multiple hydrophobic contacts that stabilize the complex. In contrast, QDK and GDR primarily achieved stable binding through electrostatic interactions with charged residues such as ARG247, GLU45, and GLU301. This stabilization likely arises from the amino group (–NH_3_^+^) or guanidino group (–C(NH_2_)_2_^+^) of positively charged residues (Lys, Arg) in the peptides forming salt bridges or electrostatic interactions with the carboxyl group (–COO^−^) of acidic residues (Glu, Asp) in the receptor.

Overall, the binding of the 10 umami peptides to the T1R1/T1R3 receptor complex was primarily mediated by hydrogen bonds, with additional stabilization provided by hydrophobic and electrostatic interactions. Among the 28 amino acid residues involved in docking, SER276, HIS145, SER146, GLU45, and ARG247 were identified as key residues for hydrogen bonding; HIS145, VAL277, and ALA302 as critical residues for hydrophobic interactions; and ARG247, GLU45, and GLU301 as essential residues for electrostatic interactions. Notably, HIS145 and ARG247 were recognized as core residues responsible for maintaining the stability of the peptide–receptor complex, consistent with previous findings [46].

## 4. Conclusions

This study systematically characterized the known flavor peptide profiles of natural and enzymatically hydrolyzed Jinhua ham extracts, revealing that bitter and umami peptides were the dominant flavor components. An integrated virtual screening pipeline combining machine learning algorithms and molecular docking was established, which efficiently identified 10 novel umami peptides (4 natural and 6 enzymatic) from over 2000 peptides, all validated by sensory evaluation and electronic tongue analysis. These peptides exhibited distinct flavor characteristics and contributed to the characteristic umami taste of Jinhua ham. Molecular docking results demonstrated that all 10 peptides had strong binding affinity to the T1R1/T1R3 umami receptor, with hydrogen bonds as the dominant interaction force (>66%), followed by hydrophobic and electrostatic interactions. HIS145 and ARG247 were identified as the key residues mediating peptide-receptor recognition. Collectively, this study expands the umami peptide repertoire of Jinhua ham and provides a robust theoretical and methodological framework for umami peptide discovery in fermented meat products.

## Figures and Tables

**Table 1:** LibDock docking results and model prediction outcomes for the 35 peptide segments.

Sequence	Resource	LibDockScore	Absolute Energy (Kcal/mol)	YYDS Pred	TPDM Pred	MRNN Pred Threshold (mmol/L)	Toxinpred3
EGY	Enz	152.502	41.901	1.000	0.911	14.383	Non-tonxin
LDAL	Nat	148.111	29.453	0.659	0.962	16.418	Non-tonxin
EQY	Enz	147.963	28.677	1.000	0.982	27.115	Non-tonxin
EAY	Enz	141.929	28.954	1.000	0.945	17.278	Non-tonxin
DGR	Enz	135.058	24.867	1.000	0.965	19.208	Non-tonxin
NHD *	Nat	131.844	29.079	0.990	0.977	12.160	Non-tonxin
LDLR *	Enz	129.786	40.898	1.000	0.986	14.221	Non-tonxin
QDK *	Enz	128.558	29.172	1.000	0.980	10.252	Non-tonxin
NEL	Nat	128.355	25.071	1.000	0.980	23.128	Non-tonxin
GDR *	Enz	126.048	25.174	1.000	0.979	8.963	Non-tonxin
LENR *	Enz	125.738	36.142	1.000	0.982	14.921	Non-tonxin
MDL	Enz	124.906	35.515	0.593	0.941	29.637	Non-tonxin
TDF *	Enz	124.439	29.918	1.000	0.980	12.993	Non-tonxin
RAE *	Nat	124.271	25.122	1.000	0.973	11.463	Non-tonxin
TEY *	Enz	124.025	28.058	1.000	0.975	12.292	Non-tonxin
ADF	Enz	123.964	39.500	1.000	0.943	17.843	Non-tonxin
TSR	Enz	122.827	37.999	1.000	0.968	12.891	Non-tonxin
EATF	Enz	114.959	35.223	1.000	0.984	32.088	Non-tonxin
VEGL *	Nat	113.956	30.730	1.000	0.977	11.831	Non-tonxin
STF	Enz	113.825	33.369	1.000	0.788	36.607	Non-tonxin
ELQR	Enz	106.581	36.161	0.760	0.986	17.398	Non-tonxin
TLEL	Nat	103.960	37.264	1.000	0.977	28.438	Non-tonxin
LENL	Nat	103.559	35.635	1.000	0.982	30.746	Non-tonxin
LEQE *	Nat	103.245	34.784	1.000	0.979	7.334	Non-tonxin
EAK	Enz	102.908	24.550	0.996	0.851	4.645	Non-tonxin
LEEA *	Enz	102.183	32.192	1.000	0.978	14.195	Non-tonxin
SELR *	Enz	101.781	37.073	1.000	0.984	5.558	Non-tonxin
TLLE	Nat	95.215	35.279	1.000	0.968	13.932	Non-tonxin
VDVL	Nat	93.542	39.313	0.970	0.901	28.940	Non-tonxin
GTG	Enz	88.904	15.276	1.000	0.916	16.858	Non-tonxin
DLR	Enz	87.454	38.007	0.898	0.968	10.371	Non-tonxin
TLVE *	Nat	83.084	37.351	1.000	0.977	10.551	Non-tonxin
GAEL	Enz	80.277	32.272	1.000	0.980	17.014	Non-tonxin
VTGL	Nat	76.654	33.344	1.000	0.740	35.395	Non-tonxin
EAR	Enz	73.873	39.107	1.000	0.946	5.699	Non-tonxin

* represents the high-confidence umami peptides.

**Table 2:** Summary of electrostatic and hydrophobic interaction forces between umami peptides and the T1R1/T1R3 umami receptor.

Name	GDR	LDLR	LEEA	LENR	LEQE	NHD	QDK	RAE	TEY	TLVE
Charge: ARG247	+		+		+	++			++	
Charge: GLU45	+			+		+	++		+	
Charge: ASP216					++		+	+		+
Charge: GLU301	++	+		+				+		
Charge: ARG64	+				+				+	
Charge: HIS145			+							
Charge: GLU148							+			
Charge: ASP190								+		
Charge: HIS278							+			
Hydrophobic: HIS145					+	+				+++
Hydrophobic: ALA302					+	+			+	+
Hydrophobic: VAL277		+	+							+
Hydrophobic: LEU308		++		+						
Hydrophobic: TYR218					+				+	
Hydrophobic: HIS278				+						+
Hydrophobic: PRO42										+
Hydrophobic: TRP72										+
Hydrophobic: LEU245		+								

Note: “+”, “++”, and “+++” denote 1, 2, and 3 binding sites, respectively.

## Data Availability

The original contributions presented in this study are included in the article/Supplementary Materials. Further inquiries can be directed to the corresponding authors.

## Supplementary Materials

The following supporting information can be downloaded at: https://www.mdpi.com/article/10.3390/foods15112019/s1, Figure S1: Profile-3D Verification Results; Figure S2: Distribution of Prediction Results Across Intervals for Three Umami Peptide Prediction Models; Table S1: Known flavor peptides of the Nat and Enz groups; Table S2: Overlap of predicted umami peptide sequences between the Nat and Enz groups; Table S3: Binding forces between ten peptides (MSG as the positive control) and the T1R1/T1R3 receptor.
