# Supplementary material for: Investigation on the Screening and Taste Mechanisms of Umami Peptides in Natural and Hydrolyzed Jinhua Ham
Source: Foods. 2026 Jun 4;15(11):2019. doi: 10.3390/foods15112019 (PMC13256520; doi:10.3390/foods15112019)

Supporting information

Table S1. Known flavor peptides of the Nat and Enz groups

Table S2. Overlap of predicted umami peptide sequences between the Nat and Enz groups.

Table S3. Binding forces between ten peptides (MSG as the positive control) and the T1R1/T1R3 receptor.

Fig. S1. Profile-3D Verification Results

Fig. S2 Distribution of Prediction Results Across Intervals for Three Umami Peptide Prediction Models

Table S1. Known flavor peptides of the Nat and Enz groups

Sequence	Group	Length	Mass	Leading razor protein	Gene Names	Intensity	Taste
DEE	Nat	3	391.1227	Q9TV62	MYH4	17228000	Umami, Sour, Salty
**DEL**	Nat	3	375.1642	A0A5G2RAA2	MYH2	92593000	Umami, Sour
EED	Nat	3	391.1227	Q9TV62	MYH4	17228000	Umami, Salty
**EEL**	Nat	3	389.1798	Q9TV62	MYH4	33935000	Umami
**EQYEEEQEAK**	Nat	10	1281.536	Q9TV62	MYH4	708860000	Umami
FAGDDAPRAVFPS	Nat	13	1348.641	P68137	ACTA1 ACTA	1667100000	Umami
FEF	Nat	3	441.19	A0A8W4FDT8	DNAH17	587190000	Umami
FPF	Nat	3	409.2002	F1SS66	MYH13	180530000	Bitter
**LDF**	Nat	3	393.19	P68137	ACTA1 ACTA	536290000	Umami
LEEL	Nat	4	502.2639	F1SS66	MYH13	61739000	Sour, Bitter
**LEF**	Nat	3	407.2056	A0A286ZPQ9	MYH6	1171600000	Umami, Bitter
LLF	Nat	3	391.2471	A0A8W4FDT8	DNAH17	60168000	Bitter
LPF	Nat	3	375.2158	A0A287AQV7	ADGRV1	629070000	Bitter
LRF	Nat	3	434.2642	A0A8W4FDT8	DNAH17	83330000	Bitter
LRL	Nat	3	400.2798	Q9TV62	MYH4	159980000	Bitter
NKF	Nat	3	407.2169	A0A287AQV7	ADGRV1	111760000	Umami, Sour, Astringent
SVW	Nat	3	390.1903	P68137	ACTA1 ACTA	63933000	Bitter
**TEF**	Nat	3	395.1693	A0A8W4FDT8	DNAH17	131670000	Umami, Kokumi
VDF	Nat	3	379.1743	A0A8W4FDT8	DNAH17	1148600000	Sour, Astringent
VEAL	Nat	4	430.2428	A0A8W4FDT8	DNAH17	23962000	Umami, Astringent
VPW	Nat	3	400.2111	A0A8W4FDT8	DNAH17	60097000	Bitter
VTY	Nat	3	381.19	A0A8W4FDT8	DNAH17	56778000	Bitter
VYP	Nat	3	377.1951	A0A287AQV7	ADGRV1	20461000	Bitter
WDL	Nat	3	432.2009	A0A5G2RKR8	APOB	171270000	Sour
YLL	Nat	3	407.242	Q9TV62	MYH4	20206000	Bitter
YPF	Nat	3	425.1951	A0A5G2RAA2	MYH2	357290000	Bitter
AGFAGDDAPR	Enz	10	975.441	P68137	ACTA1 ACTA	7269300000	Umami, Sweet, Kokumi
AVL	Enz	3	301.2002	A0A287AZ59	ABCA13	24017000	Bitter
DAGVIAGLNVLR	Enz	12	1196.688	A0A900R1F0	HSPA1L	11981000	Umami, Sweet, Kokumi
**DEL**	Enz	3	375.1642	A0A287AZ59	ABCA13	44083000	Umami, Sour
DGF	Enz	3	337.1274	A0A287AZ59	ABCA13	12252000	Umami, Astringent
EAF	Enz	3	365.1587	A0A287AZ59	ABCA13	353470000	Umami, Sweet
EDF	Enz	3	409.1485	A0A287AZ59	ABCA13	8737900	Umami
**EEL**	Enz	3	389.1798	A0A287AZ59	ABCA13	10992000	Umami
EELR	Enz	4	545.2809	A0A287AZ59	ABCA13	2951900	Umami, Salty
ELL	Enz	3	373.2213	A0A287AZ59	ABCA13	26937000	Bitter
**EQYEEEQEAK**	Enz	10	1281.536	Q9TV62	MYH4	291400000	Umami
FAGDDAPR	Enz	8	847.3824	P68137	ACTA1 ACTA	230210000	Umami, Sweet, Sour, Astringent
FPK	Enz	3	390.2267	A0A8W4FHH8	OBSCN	40516000	Bitter
GLL	Enz	3	301.2002	A0A287AZ59	ABCA13	1164500000	Bitter

GLY	Enz	3	351.1794	A0A287AZ59	ABCA13	61583000	Bitter
**LDF**	Enz	3	393.19	A0A287AZ59	ABCA13	102510000	Umami
LDL	Enz	3	359.2056	A0A8W4FHH8	OBSCN	81277000	Umami, Bitter
**LEF**	Enz	3	407.2056	A0A8W4FHH8	OBSCN	71712000	Umami, Bitter
LEL	Enz	3	373.2213	A0A287AZ59	ABCA13	33052000	Bitter
LEQLL	Enz	5	614.3639	A0A287AZ59	ABCA13	2793300	Bitter
LGL	Enz	3	301.2002	A0A287AZ59	ABCA13	5401100	Bitter
LGLR	Enz	4	457.3013	A0A8W4FHH8	OBSCN	109970000	Umami, Bitter
LLF	Enz	3	391.2471	A0A287AZ59	ABCA13	2055600	Bitter
LLL	Enz	3	357.2628	A0A287AZ59	ABCA13	300090000	Bitter
LLLL	Enz	4	470.3468	A0A287AZ59	ABCA13	46187000	Bitter
LLLLR	Enz	5	626.4479	A0A287AZ59	ABCA13	40216000	Bitter
LPF	Enz	3	375.2158	A0A287AZ59	ABCA13	128500000	Bitter
LPL	Enz	3	341.2315	A0A287AZ59	ABCA13	20931000	Bitter
LQL	Enz	3	372.2373	A0A287AZ59	ABCA13	30229000	Bitter
LRL	Enz	3	400.2798	A0A287AZ59	ABCA13	23987000	Bitter
LVL	Enz	3	343.2471	A0A287AZ59	ABCA13	142760000	Bitter
PTVEVDLH	Enz	8	908.4604	A0A5G2R7R6	ENO3	29924000	Umami, Sour
RRR	Enz	3	486.3139	A0A287AZ59	ABCA13	30516000	Bitter
**TEF**	Enz	3	395.1693	A0A287AZ59	ABCA13	20423000	Umami, Kokumi
TPE	Enz	3	345.1536	A0A8W4FHH8	OBSCN	5308000	Umami
VDF	Enz	3	379.1743	A0A287AZ59	ABCA13	38117000	Sour, Astringent
VEL	Enz	3	359.2056	A0A8W4FHH8	OBSCN	3077200	Umami
VEV	Enz	3	345.19	A0A8W4FHH8	OBSCN	5792000	Umami
VNVDEVGGEALGR	Enz	13	1313.658	A0A5G2QRW3		364980000	Umami, Sour
VTY	Enz	3	381.19	A0A8W4FHH8	OBSCN	9248800	Bitter

***Leading razor protein：The identifier of the top-ranked protein in the proteome corresponding to the peptide；Gene Names：The gene name corresponding to the top-ranked protein in the proteome in the database；　Intensity：Peak intensity；The sequence displayed in bold is the intersection of umami peptides from Nat and Enz groups**

Table S2. Overlap of predicted umami peptide sequences between the Nat and Enz groups

Sequence	Length	Mass	Leading razor protein	Gene Names	Intensity	MRNN_Pred (mmol/L)	TPDM_Pred	YYDS_Pred	Toxinpred 3
VVDL	4	444.2584	A0A287AZ59	ABCA13	73526000	24.3763	0.985	0.9701	Non-Toxin
SYVGDEAQSK	10	1082.488	P68137	ACTA1 ACTA	2E+08	0.79071	0.978	1	Non-Toxin
YVGDEAQSKR	10	1151.557	P68137	ACTA1 ACTA	27552000	2.356909	0.978	1	Non-Toxin
LGEQIDNLQR	10	1184.615	Q9TV62	MYH4	54250000	4.457671	0.978	1	Non-Toxin
ITLSQVGDVLR	11	1199.687	A0A8D0VIH6	MYL1	7.16E+08	2.328226	0.978	1	Non-Toxin
TEELEEAKKK	10	1203.635	Q9TV62	MYH4	1.73E+08	6.802295	0.978	1	Non-Toxin
DLQHRLDEAE	10	1224.574	Q9TV62	MYH4	8879600	4.64487	0.978	1	Non-Toxin
KNNLGELINTL	11	1227.682	D0G7F6	TPI1	83634000	24.8694	0.978	1	Non-Toxin
RTEELEEAKK	10	1231.641	Q9TV62	MYH4	1.54E+08	8.445992	0.978	1	Non-Toxin
ELEGEVESEQK	11	1275.583	Q9TV61	MYH1	1.08E+09	7.91829	0.978	1	Non-Toxin
LQDLVDKLQSK	11	1285.724	Q9TV62	MYH4	2.3E+09	4.607503	0.978	1	Non-Toxin
EQYEEEQESK	10	1297.531	F1SS64	MYH2	85965000	5.498632	0.978	1	Non-Toxin
SGGTTMYPGIADR	13	1324.608	P68137	ACTA1 ACTA	3.89E+08	1.871656	0.985	1	Non-Toxin
EVEDLMLDVER	11	1346.639	Q9TV62	MYH4	35830000	3.174961	0.975	1	Non-Toxin
PLNETVVGLYQK	12	1359.74	Q9TV62	MYH4	17071000	15.99071	0.978	1	Non-Toxin
LQHELEEAEER	11	1381.647	Q9TV62	MYH4	6.72E+08	5.902403	0.979	1	Non-Toxin
GKQAFTQQIEEL	12	1390.709	Q9TV62	MYH4	12755000	16.19126	0.979	1	Non-Toxin
IGMESAGIHETTY	13	1407.634	P68137	ACTA1 ACTA	5.78E+08	18.08455	0.978	1	Non-Toxin
ELEGEVESEQKR	12	1431.684	Q9TV61	MYH1	8.09E+09	5.654025	0.978	1	Non-Toxin
RLQDLVDKLQSK	12	1441.825	Q9TV62	MYH4	31498000	17.14632	0.977	1	Non-Toxin
EQYEEETEAKAE	12	1454.605	P79293	MYH7	5130200	3.212796	0.978	1	Non-Toxin
DLQHRLDEAEQL	12	1465.716	Q9TV62	MYH4	1.84E+08	12.10734	0.978	1	Non-Toxin
RLQDLVDKLQLK	12	1467.877	P79293	MYH7	1774800	23.18077	0.977	1	Non-Toxin
TEELEEEIEAER	12	1475.663	Q9TV62	MYH4	1.68E+09	7.531635	0.979	1	Non-Toxin

Table S3. Binding forces between ten peptides (MSG as the positive control) and the T1R1/T1R3 receptor.

Seq	-CDOCKER_ENERGY	-CDOCKER_INTERACTION_ENERGY	HydrogenBond Total	Charge Total	Hydrophobic Total
QDK	96.3489	75.173	12	5	0
LEQE	94.9572	77.5133	18	4	3
LDLR	91.6731	79.9905	13	1	4
LENR	90.7402	86.7166	18	2	2
NHD	89.8272	62.5343	15	3	2
LEEA	83.2064	56.6713	11	2	1
GDR	80.6051	73.0974	17	5	0
TLVE	80.4206	78.0647	18	1	8
TEY	78.7666	70.4045	12	4	2
RAE	78.4521	72.8492	13	3	0
MSG	22.2749	46.5836	9	2	5

Fig. S1. Profile-3D Verification Results

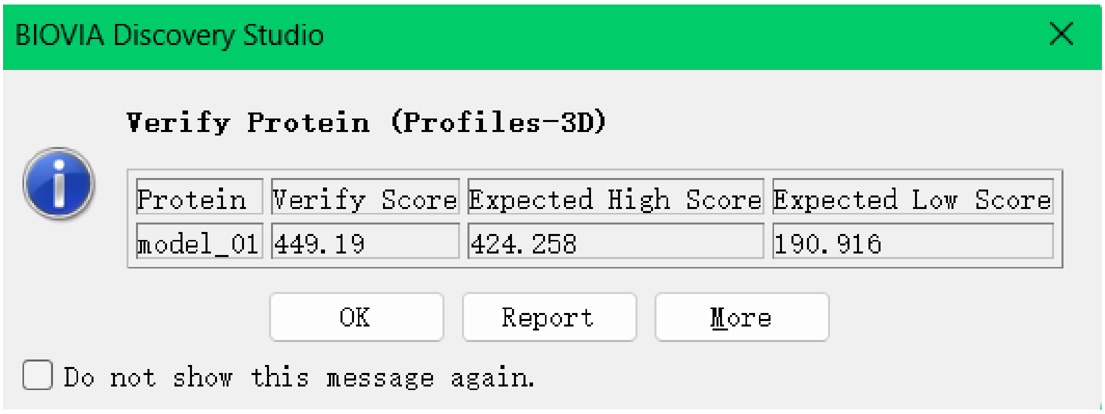

Fig. S2. Kernel density estimation (KDE) plots of umami prediction scores for the 35 candidate peptides across YYDS, TPDM, and MRNN models.

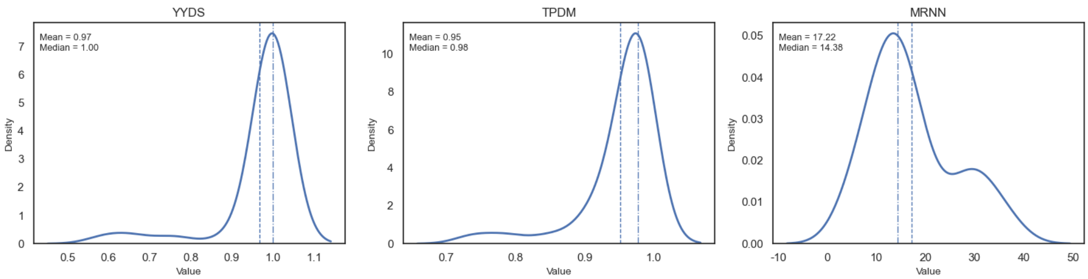

Supplement: Supplementary file 1 [file foods-15-02019-s001.zip › foods-4317610-supplementary.pdf]
